# Genome-wide study of C2H2 zinc finger gene family in *Medicago truncatula*

**DOI:** 10.1186/s12870-020-02619-6

**Published:** 2020-08-31

**Authors:** Zhicheng Jiao, Liping Wang, Huan Du, Ying Wang, Weixu Wang, Junjie Liu, Jinhang Huang, Wei Huang, Liangfa Ge

**Affiliations:** 1 Department of Grassland Science, College of Forestry and Landscape Architecture, South China Agricultural University, Guangzhou, 510642 Guangdong China; 2 Guangdong Engineering Research Center for Grassland Science, Tianhe, 483 Wushan Road, Guangzhou, 510642 Guangdong China; 3 Guangdong Key Laboratory for Innovative Development and Utilization of Forest Plant Germplasm, South China Agricultural University, Guangzhou, 510642 Guangdong China; 4 State Key Laboratory for Conservation and Utilization of Subtropical Agro-Bioresources, South China Agricultural University, Guangzhou, 510642 Guangdong China; 5 College of Life Sciences, South China Agricultural University, Guangzhou, 510642 Guangdong China

**Keywords:** C2H2, Zinc finger, Local gene duplication, Gene family, EAR motif, Expression

## Abstract

**Background:**

C2H2 zinc finger proteins (C2H2 ZFPs) play vital roles in shaping many aspects of plant growth and adaptation to the environment. Plant genomes harbor hundreds of C2H2 ZFPs, which compose one of the most important and largest transcription factor families in higher plants. Although the C2H2 ZFP gene family has been reported in several plant species, it has not been described in the model leguminous species *Medicago truncatula*.

**Results:**

In this study, we identified 218 C2H2 type ZFPs with 337 individual C2H2 motifs in *M. truncatula*. We showed that the high rate of local gene duplication has significantly contributed to the expansion of the C2H2 gene family in *M. truncatula*. The identified ZFPs exhibit high variation in motif arrangement and expression pattern, suggesting that the short C2H2 zinc finger motif has been adopted as a scaffold by numerous transcription factors with different functions to recognize cis-elements. By analyzing the public expression datasets and quantitative RT-PCR (qRT-PCR), we identified several C2H2 ZFPs that are specifically expressed in certain tissues, such as the nodule, seed, and flower.

**Conclusion:**

Our genome-wide work revealed an expanded C2H2 ZFP gene family in an important legume *M. truncatula*, and provides new insights into the diversification and expansion of C2H2 ZFPs in higher plants.

## Background

Zinc finger proteins (ZFPs) have evolved and diversified into a massive transcription factor family in the plant kingdom [1, 2]. ZFPs contain one to a few zinc fingers, which are approximately 23 to 30 amino acids in length and have several cysteine and histidine residues. The cysteine and histidine residues in the motif coordinately bind one or more zinc ions through hydrogen bonds, forming a stabilized finger-like structure that can interact with nucleic acid sequences to regulate target gene expression. Based on the combination of cysteine and histidine residues, ZFPs can be classified into different types, such as C2H2, C2C2, C2HC, and C3H [3–6].

The C2H2 type zinc finger is one of the most common motifs in ZFPs, and it can be represented by the sequence X2-Cys-X2,4-Cys-X12-His-X3,4,5-His (X indicates any amino acid residue and numbers represent residue quantity) [7, 8]. This approximately 23–30-amino-acid-motif contains two β-sheet strands and one *α*-helix in the N-terminus and C-terminus, respectively, which fold around the center zinc ion to establish a stabilized simple structure that binds to the major groove of the target DNA sequence [8]. The number of C2H2 zinc fingers in ZFPs is variable, ranging from one to even dozens, thus providing the flexible affinity to bind nucleic acid [7, 9]. With the ability to interact with the DNA segment, C2H2 ZFPs mainly play a role in controlling the transcription of target genes, serving as typical transcription factors. Interestingly, the ethylene-responsive element binding factor-associated amphiphilic repression motif (EAR), which binds to the transcriptional corepressor TOPLESS (TPL) and suppresses the expression of downstream genes, is often found in C2H2 ZFPs, thereby enabling transcriptional repression functions of C2H2 ZFPs. In addition, some C2H2 ZFPs can bind RNA or other proteins to participate in RNA metabolism, gene expression or protein activity regulation [9–13].

C2H2 ZFP was first reported in the African clawed frog (*Xenopus laevis*) transcription factor IIIA (TFIIIA) protein, which consists of nine repetitive tandem C2H2 motifs [14]. In higher plant species, hundreds of C2H2 ZFPs were found in sequenced genomes [1, 12, 15, 16], including some that have been functionally characterized. C2H2 ZFPs exhibit a broad range of roles in regulating many aspects of plant growth and development, phytohormone signaling, and abiotic and biotic stress responses [13, 17–22].

It has been proposed that C2H2 motifs can be generally classified into two types: the Q-type and the C-type. Q-type C2H2 has the core sequence ‘QALGGH’, where ‘H’ is the first histidine of the C2H2 motif. Several Q-type C2H2 ZFPs have been well studied, including *SUPERMAN* (*SUP*) in Arabidopsis, *DROUGHT AND SALT TOLERANCE 1* (*DST1*) in rice and *PALMATE-LIKE PENTAFOLIATA1* (*PALM1*) / *INHIBITOR OF RUST GERM TUBE DIFFERENTATION1* (*IRG1*) in *M. truncatula* [17, 18, 21, 23, 24]. This type of C2H2 zinc finger was reported to be specific to plants, whereas C-type proteins presented in all organisms and did not contain conserved core sequences [1, 12, 15, 16, 25, 26]. Besides the Q-type and C-type C2H2 ZFPs, some recent genome-wide studies revealed the presence of new types of C2H2s in plant species, such as the modified Q-type (QM-type) and Z-type C2H2 motifs [15, 26, 27].

*Medicago truncatula* (also called barrel medic) is a model legume species that is phylogenetically close to the forage species alfalfa (*Medicago sativa*). In *M. truncatula*, only few C2H2 ZFPs have been characterized, such as *PALM1* / *IRG1* and *REGULATOR OF SYMBIOSOME DIFFERENTIATION* (*RSD*) [23, 24, 28, 29], both of which have a single C2H2 motif at the N-terminus and an EAR motif at C-terminus. *PALM1*/*IRG1* plays an important role in determining the *M. truncatula* compound leaf pattern and epicuticular wax metabolism [23, 24, 29, 30], whereas *RSD* controls symbiosome differentiation during nodule development [28].

Most C2H2 ZFPs in *M. truncatula* are not yet characterized, and a genome-wide sequence and expression analysis of C2H2 ZFPs is lacking. In this study, a total of 218 C2H2 type ZFPs were identified in the *M. truncatula* genome, with the majority of them containing only a single C2H2 motif. We systematically classified C2H2 motifs and ZFPs, and analyzed the motif arrangement of the identified genes. Our results showed that, except for the conserved motifs, the C2H2 ZFPs displayed high sequence variation among the family members, demonstrating the functional diversification and supporting the notion that the C2H2 motif has been adopted by the plant genome to provide a versatile scaffold for proteins to recognize targeted DNA sequences. Using sequence analysis and a homolog searching-based method, we identified many C2H2 ZFPs that showed high similarity to well-characterized ZFPs in other plant species, such as indeterminate-domain (IDD) genes. Consistent with the sequence variation, microarray-based expression analysis showed highly variable expression patterns of the C2H2 ZFPs, further supporting the functional diversification of the genes in this family. Expression analysis by quantitative RT-PCR (qRT-PCR) also confirmed a few C2H2 ZFPs that were specifically expressed in certain tissues, providing valuable candidates for further C2H2 gene function studies. Our work identified and classified a big family of C2H2 type ZFPs in *M. truncatula*, and performed gene structure and expression analysis for the identified ZFPs. The picture of the gene family presented in this work gives insights into the expansion and diversification of C2H2 ZFPs in *M. truncatula*, and will help to understand the roles of C2H2 ZFPs in this model legume.

## Results

### Overview of C2H2 ZFPs in the *M. truncatula* genome

**Fig. 1:**
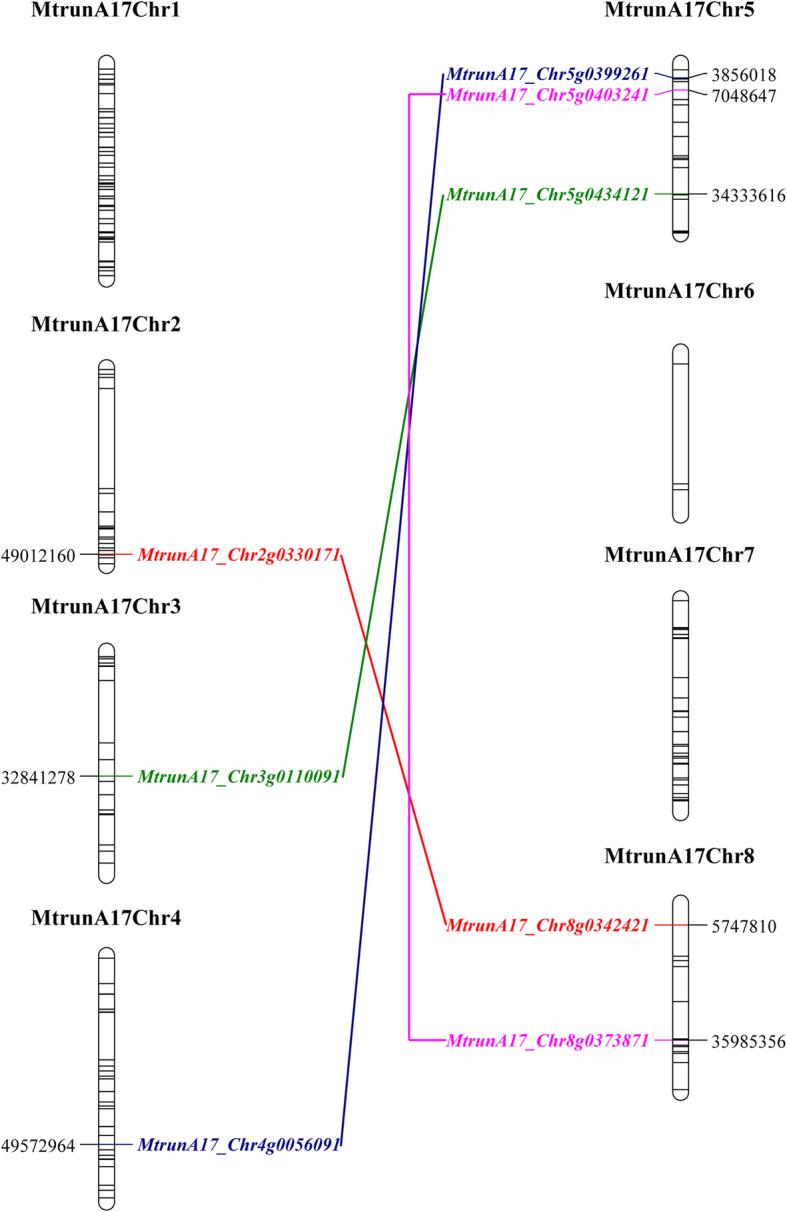
MapChart diagram illustrating the distribution of C2H2 ZFPs in the *M. truncatula* genome. The black lines within the chromosomes indicate C2H2 ZFPs. The line linking two C2H2 ZFPs represents a pair of C2H2 ZFPs resulting from WGD. Different colors of the line and gene ID indicate different pairs of C2H2 ZFPs from WGD

There are 112 C2H2 type ZFPs listed in the Medicago transcription factor database (http://planttfdb.cbi.pku.edu.cn/index.php?sp=Mtr). Given the large number of C2H2 ZFPs reported in other species [15, 16, 27], for instance, 176 and 321 C2H2 ZFPs in the Arabidopsis and soybean genome, respectively [16, 27], we thought that there should be more C2H2 ZFPs in the *M. truncatula* genome. To date, very few C2H2 ZFPs have been reported in *M. truncatula*. Thus, a literature survey did not return much useful information regarding the C2H2 ZFP family in *M. truncatula*. To extensively identify C2H2 ZFPs, we employed a combined strategy and searched the genome databases MtrunA17r5.0 (https://medicago.toulouse.inra.fr/MtrunA17r5.0-ANR) and Mt4.0v2 (http://www.medicagogenome.org) [31–33]. Based on the reported C2H2 motif sequences pattern, we developed a custom script using the Regular Expression module in the programming language Python. As a result, 272 candidate C2H2 ZFPs containing 381 C2H2 motifs were found. To eliminate potential false positives, the candidates were further scanned in the Prosite protein database and PFAM platforms for confirmation. The candidates that passed the C2H2 motif scan in Prosite and PFAM platforms or showed significant similarity to *Arabidopsis* or rice C2H2 ZFPs were considered to be *M. truncatula* C2H2 type ZFPs. This step eliminated 55 candidates and resulted in 217 C2H2 ZFPs containing 328 C2H2 motifs in *M. truncatula* (Fig. 1, Additional file 1, Additional file 2 and Additional file 3).

TFIIIA is the ZFP that contain the most C2H2 motifs in plant species [14, 16, 34]. An initial examination of the identified 217 ZFPs found that TFIIIA was not present in the *M. truncatula* latest genome (Jemalong A17). We doubt that it may be caused by sequencing or annotation error. Using the genome sequence of another cultivar R108 (http://www.medicagohapmap.org/downloads/r108) and Affymetrix microarray probeset data, we identified and manually sequenced TFIIIA homolog in *M. truncatula* (described below).

In summary, we identified a total of 218 C2H2 ZFPs containing 337 C2H2 motifs (Fig. 1, Additional file 1, Additional file 2 and Additional file 3), far exceeding the genes listed in the plant transcription factor database. The C2H2 ZFPs identified in *M. truncatula* displayed variations in length and molecular weight, ranging from less than 100 to more than 1700 amino acids, and 7.3 to 197 kilodaltons (kDa), respectively. However, a large portion of the family (182 of the total of 218 ZFPs) was quite short (less than 500 amino acids). The identified C2H2 ZFPs also showed a wide range of the isoelectric point (PI) from 4.14 to 9.92, indicating the diversified physicochemical properties of the proteins. Many C2H2 ZFPs have splice variants, further adding the protein diversity of the gene family (Additional file 1).

According to the MtrunA17r5.0 and Mt4.0v2 genome databases, all C2H2 ZFPs are found on the chromosomes. Chromosome 1 harbored the most (59 C2H2 ZFPs), representing approximately 27% of the whole family, followed by chromosome 7 (36 C2H2 ZFPs) and chromosome 4 (35 C2H2 ZFPs). Chromosome 6 and chromosome 8 contained only 3 and 14 C2H2 ZFPs, respectively (Fig. 1, Additional file 4).

In *M. truncatula* C2H2 ZFP gene family, 46 local duplications containing two to six C2H2 ZFPs were found (Additional file 5). For instance, the neighboring genes *MtrunA17_Chr3g0086321/Medtr3g023750* and *MtrunA17_Chr3g0086331/Medtr3g023760* both encode C2H2 type ZFPs; a 6.5 kb fragment on chromosome 1 from 14.30 M to 14.31 M contains three C2H2 type ZFPs: *MtrunA17_Chr1g0164501*, *MtrunA17_Chr1g0164511* and *MtrunA17_Chr1g0164521* (Additional file 5). The local duplication of C2H2 ZFPs significantly increased the total number of C2H2 ZFPs in *M. truncatula*, and provided the potential for the subfunctionalization and diversification of C2H2 ZFPs.

As a papilionoids species, *M. truncatula* underwent a whole-genome duplication (WGD) 58 million years ago (Myr) [35–37]. WGD gave rise to gene pairs, which are located in syntenic genome regions and have been detected by genome-level analysis [35]. By gene position analysis, MCScanX analysis, and comparison with a previous dataset [38], we found that 4 pairs of C2H2 ZFPs derived from WGD (Fig. 1, Additional file 6). In agreement with the duplication, the C2H2 motif type and arrangement were identical between the two paralogs from the gene pairs (Additional file 6).

To gain basic phylogenetic insights into the relationship of C2H2 ZFPs in *M. truncatula*, we attempted to perform sequence alignment and reconstruct the phylogenetic tree for the C2H2 ZFPs. However, the sequence similarity analysis indicated that, except for close homologs or subsets such as MtrunA17_Chr8R0156030/Medtr8g466760 and MtrunA17_Chr3g0081131/Medtr3g011990 that share a significant similarity, members of the C2H2 ZFP family are rather different in sequence and structure, as less than 15% similarity was detected for more than 97% of genes, suggesting the functional diversification of C2H2 ZFPs (Additional file 7).

### Identification of TFIIIA homolog in the *M. truncatula* genome

**Fig. 2:**
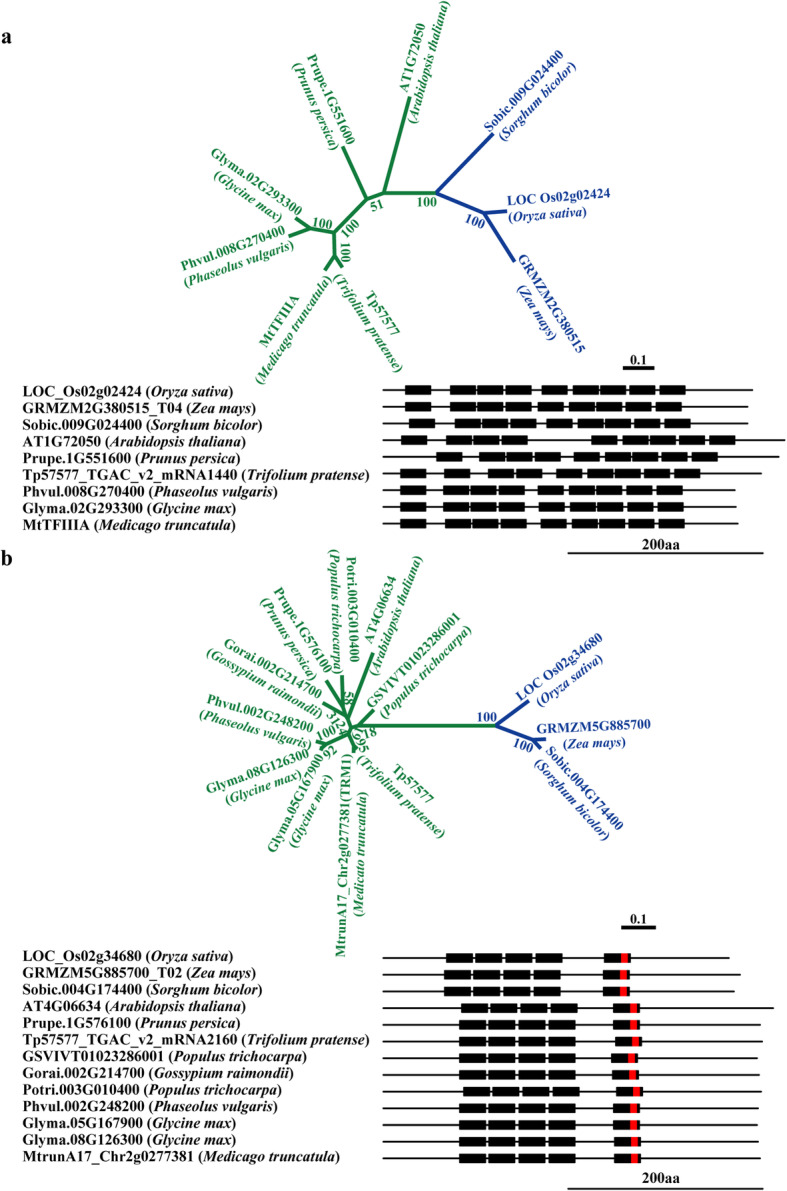
Phylogenetic analysis and motif arrangement of TFIIIA and TRM1 homologs. The top part of (**a**) and (**b**) were the unrooted maximum-likelihood tree of TFIIIA and TRM1 homologs, respectively. The blue and green branches indicated monocot and dicot plants respectively. The bottom part of (**a**) and (**b**) are motif arrangements of TFIIIA and TRM1, respectively. The black line represents the protein sequence. The black rectangle indicates the C2H2 motif, and the red rectangle indicates the EAR motif

The *Arabidopsis* homolog of *TFIIIA* has been previously reported to regulate the transcription of 5S RNA genes [16, 34]. The *M. truncatula* TFIIIA homolog was not found by BLAST search using *Arabidopsis* TFIIIA as the query protein against both Jemalong A17 Mt4.0v2 and MtrunA17r5.0 genome sequence, probably because that the *M. truncatula TFIIIA* gene model has not yet been predicted in the genome sequence. A BLAST search against the *M. truncatula* Affymetrix Microarray probesets found that probeset *Mtr.14440.1.S1_at*, which corresponds to a genome region located on scaffold MWMB01000024 of *M. truncatula* R108 genome (http://www.medicagohapmap.org/downloads/r108), is highly similar to *AtTFIIIA*, suggesting that the *TFIIIA* homolog was also present in the *M. truncatula* R108 genome. BLAST searches in other plant species also revealed TFIIIA homologs in other plant genomes. To confirm the existence of TFIIIA in Medicago R108 genome, we amplified and sequenced the coding region of *M. truncatula* TFIIIA homolog. The identified *M. truncatula* TFIIIA homolog is consistent with the Hidden Markov Model (HMM) profile built with TFIIIA homologs from other plants by HMMER (http://hmmer.org/), confirming the identification of TFIIIA homolog in *M. truncatula*. Gene sequences analysis indicated that, similar to Arabidopsis TFIIIA, *M. truncatula* and other plant TFIIIA homologs consisted of nine C2H2 zinc fingers (Fig. 2a). Phylogenetic and sequence similarity analysis showed that all plant TFIIIAs shared a high sequence identity (Fig. 2a), suggesting a conserved function in regulating the transcription of 5S RNA genes. In *Arabidopsis* TFIIIA, the 2–4 and 5–9 fingers were arranged in two tandem finger arrays, whereas the first C2H2 zinc finger was relatively separated at the N-terminus. This pattern was also observed in other plant TFIIIA homologs, though the link sequence between the first and second finger array was a little shorter, especially in monocot plants (Fig. 2a). Nevertheless, this similar motif arrangement pattern suggests a conserved evolutional ancestor of plant TFIIIAs.

### Analysis of TRM1 homolog in the *M. truncatula* genome

*TRM1* was first cloned and characterized in the C4 plant maize (*Zea mays*), in which TRM1 binds to the *rbcS-m3* gene and plays a transcriptional repressor role [39]. The *TRM1* homolog has also been identified in Arabidopsis [16]. Like TFIIIA, TRM1 was not initially found by BLAST search in the *M. truncatula* Mt4.0v2 genome database using either the Arabidopsis or maize TRM1 protein sequence as the query. However, a BLAST search against *M. truncatula* MtrunA17r5.0 genome database found that MtrunA17_Chr2g0277381 shows high similarity to Arabidopsis TRM1. We found that an Affymetrix probeset *Mtr.25069.1.S1_s_at* showed high similarity to TRM1 protein, suggesting that TRM1 was also present in the *M. truncatula* genome. To confirm the existence of *TRM1* in *M. truncatula*, we amplified and sequenced the *M. truncatula TRM1* homolog. Like maize TRM1, the *M. truncatula* TRM1 homolog contained five C2H2 motifs (Fig. 2b). The C2H2 motifs of TRM1 could be separated into two classes based on the linker length (Fig. 2b). The first four motifs formed a tandem repeat separated by 29 or 30 linker residues. The fifth motif was 65 residues away from the fourth motif (Fig. 2b). HMM analysis indicated that the *M. truncatula* TRM1 is consistent with the HMM profile built with known TRM1 homologs by HMMER. A sequence search in other plant species also identified putative TRM1 homologs that shared a high sequence identity and conserved phylogenetic relationship, suggesting a conserved role of TRM1 in plant species (Fig. 2b). Interestingly, the N-terminus of TRM1 from monocot species was a little shorter than its counterparts from dicot species. In rice, maize, and sorghum, the N-terminus, which started from the first residue to the first C2H2 motif, was 61 or 63 amino acids in length, whereas it ranged from 76 to 80 amino acids in dicot plants (Fig. 2b).

Surprisingly, a putative EAR motif-like sequence, ‘LKLHLK’, was found within the fifth C2H2 motif of *M. truncatula* TRM1 and TRM1 homologs in other plant species, including Arabidopsis, soybean, rice, maize, and sorghum (Fig. 2b).

### C2H2 motifs in *M. truncatula*

The 218 C2H2 type ZFPs identified in the *M. truncatula* genome contained 337 individual C2H2 motifs. We aligned *M. truncatula* C2H2 motifs and classified them into five major groups based on the conserved core sequences within the C2H2 domains (Additional file 8).

Q-type C2H2s were found in 93 motifs from 71 genes. Q-type C2H2 had the core sequence ‘QALGGH’, where ‘H’ is the first histidine of the C2H2 motif. This type of C2H2 zinc finger has been previously reported to be specific to plants [1, 12, 15, 16, 25]. In addition to the core sequence, the three residues between the two histidines also showed the high conservation in certain Q-type motifs. Regarding the residues between the two histidines, we further subclassified Q-type C2H2s into six subgroups, including 28 motifs with the consensus ‘QNA’ between the two histidines (Q1 subgroup), 22 motifs with ‘(K/R)AS’ (Q2 subgroup), 12 motifs with ‘MR(R/K)’ (Q3 subgroup), 10 motifs with ‘MN(I/V)’ (Q4 subgroup), 10 motifs with ‘KR(C/S)’ (Q5 subgroup), and 11 motifs without the consensus sequence between the two histidines (Q6 subgroup) (Additional file 8).

In some C2H2 motifs, the core sequences of the Q-type motif were slightly or moderately modified, such as ‘RALGGH’ or ‘QGLGGH’. These C2H2s have also been reported in other plant species, and herein were designated as the Q-Modified (QM) type. In the *M. truncatula* genome, 57 QM-type C2H2 motifs were identified from 45 ZFPs. Based on the pattern of the core sequences and the methods used in a previous report [15], we classified the QM-type C2H2 motifs into nine subgroups (Additional file 8).

**Fig. 3:**
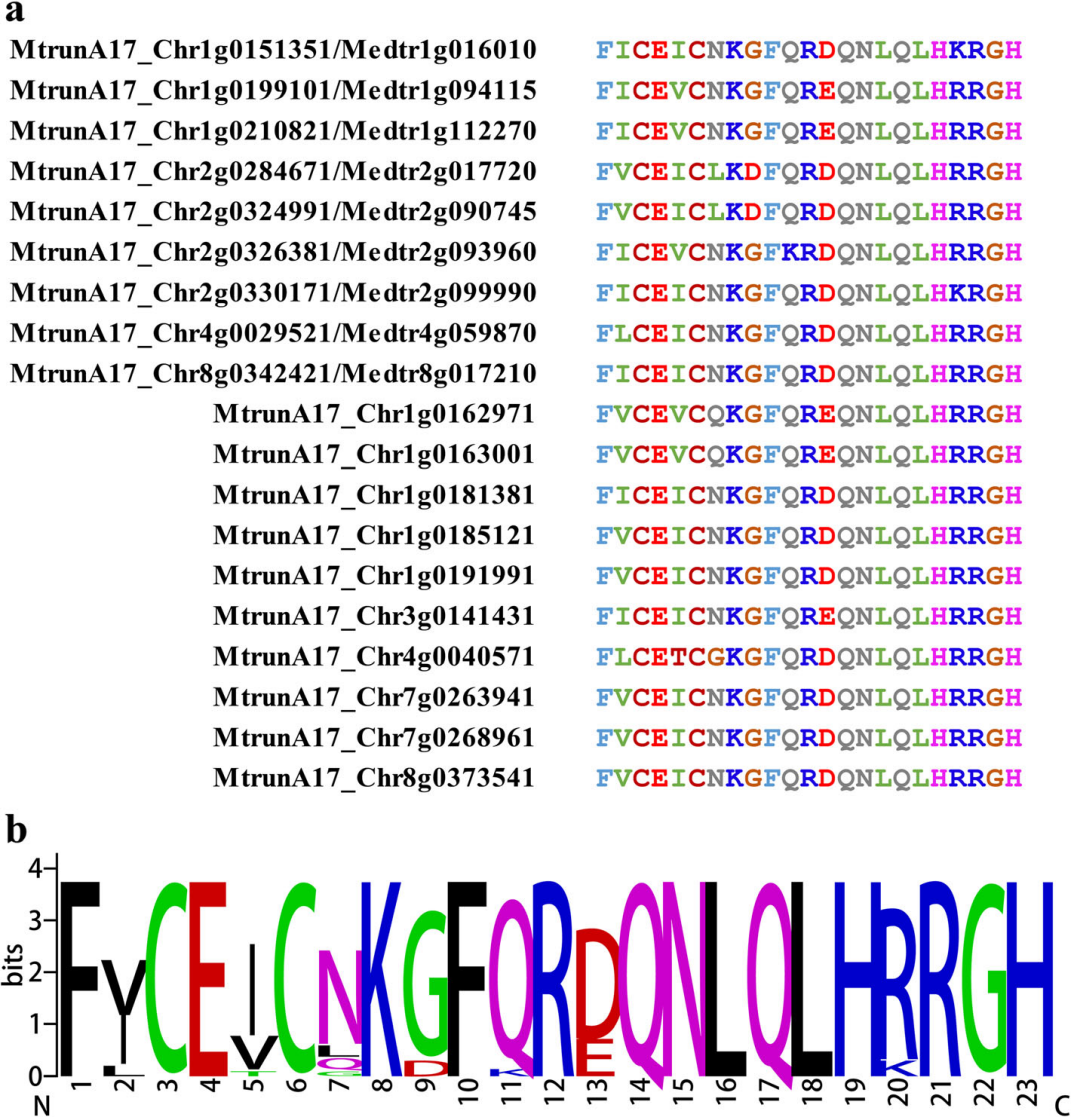
IDD-type C2H2 motifs. **a** Alignment of IDD-type C2H2 motifs from *M. truncatula*. **b** Signature of IDD-type C2H2 motifs

In nineteen ZFPs, a new type of conserved C2H2 zinc motif was found. Starting with phenylalanine (F), this type motif was even more conserved than the Q-type. Of a total of 23 amino acids, 16 were identical across all the genes (Fig. 3, Additional file 8). In Arabidopsis, this type of C2H2s was found in indeterminate-domain (IDD) genes, such as IDD10/JKD (AT5G03150), BALDIBIS (AT3G45260), IDD5 (AT2G02070) and IDD7 (AT1G55110). The first IDD gene ID1 was cloned in maize where it controlled the transition to reproductive growth [40]. The IDD family in plants consists of multiple members, with different roles in regulating plant growth or metabolism. Sequence comparison indicated that *M. truncatula* ZFPs containing this type of C2H2s demonstrated high similarity to Arabidopsis IDDs. For instance, MtrunA17_Chr8g0342421/Medtr8g017210, which contains this type of C2H2 zinc finger, shows a high similarity to Arabidopsis IDD11 (AT3G13810). Because of the prevailing presence in IDD genes, this type of zinc finger was named IDD-type C2H2. Although IDD genes have not been previously reported in *M. truncatula*, the identification of IDD-type C2H2 confirmed the presence and putative conserved function of this important gene family in *M. truncatula*.

Z-type motifs represent C2H2s that display highly conserved core sequences and do not belong to Q, QM, and IDD-type C2H2s. We identified 41 C2H2 motifs from 25 ZFPs, which were classified as Z-type (Additional file 8). Based on the pattern of the core sequences within the C2H2 motifs, Z-type C2H2s were further classified into eight subgroups, with 2 to 15 individual motifs in each subgroup (Additional file 8). Previous reports grouped the C2H2s that did not contain any conserved core sequences to C-type [1, 15, 26]. In this work, we identified 127 C-type C2H2s.

### Classification of C2H2 ZFPs in *M. truncatula*

The C2H2 ZFPs identified in *M. truncatula* were naturally classified into groups based on the C2H2 zinc finger numbers in ZFPs. Among the 218 C2H2 ZFPs, 151 genes had a single C2H2 motif, representing 69% of the family. The dominant abundance of single C2H2 motif genes in the *M. truncatula* genome is consistent with the ZFP family in other plant species [16], and highlights the difference in the C2H2 ZFP family between plant and animal genomes, wherein single C2H2 motif genes are rarely found [7]. Among the 67 ZFPs with multiple C2H2s, 38 genes had two zinc fingers, 16 had three zinc fingers, 9 had four zinc fingers, one (TRM1) had five zinc fingers, two have six and one (TFIIIA) had nine zinc finger C2H2 motifs (Additional file 9).

### ZFPs with a single C2H2 motif in *M. truncatula*

The majority of C2H2 ZFPs found in the *M. truncatula* genome contained only one C2H2 motif. Among a total of 218 ZFPs found, 151 had only one C2H2 zinc finger motif. The EAR motif connects transcription suppressor TPL/TPL-like proteins, and thus enables transcription repression functions of the coding genes. The EAR motif is often found in ZFP genes. Based on the presence of the EAR motif, we categorized the 151 single C2H2 ZFPs into three subgroups, i.e., ZFPs without an EAR, ZFPs with one EAR and ZFPs with multiple EARs. Among 151 single C2H2 ZFPs, 74 contained no EAR, 60 contained one EAR, and 17 contained multiple EAR motifs (Additional file 10).

**Fig. 4:**
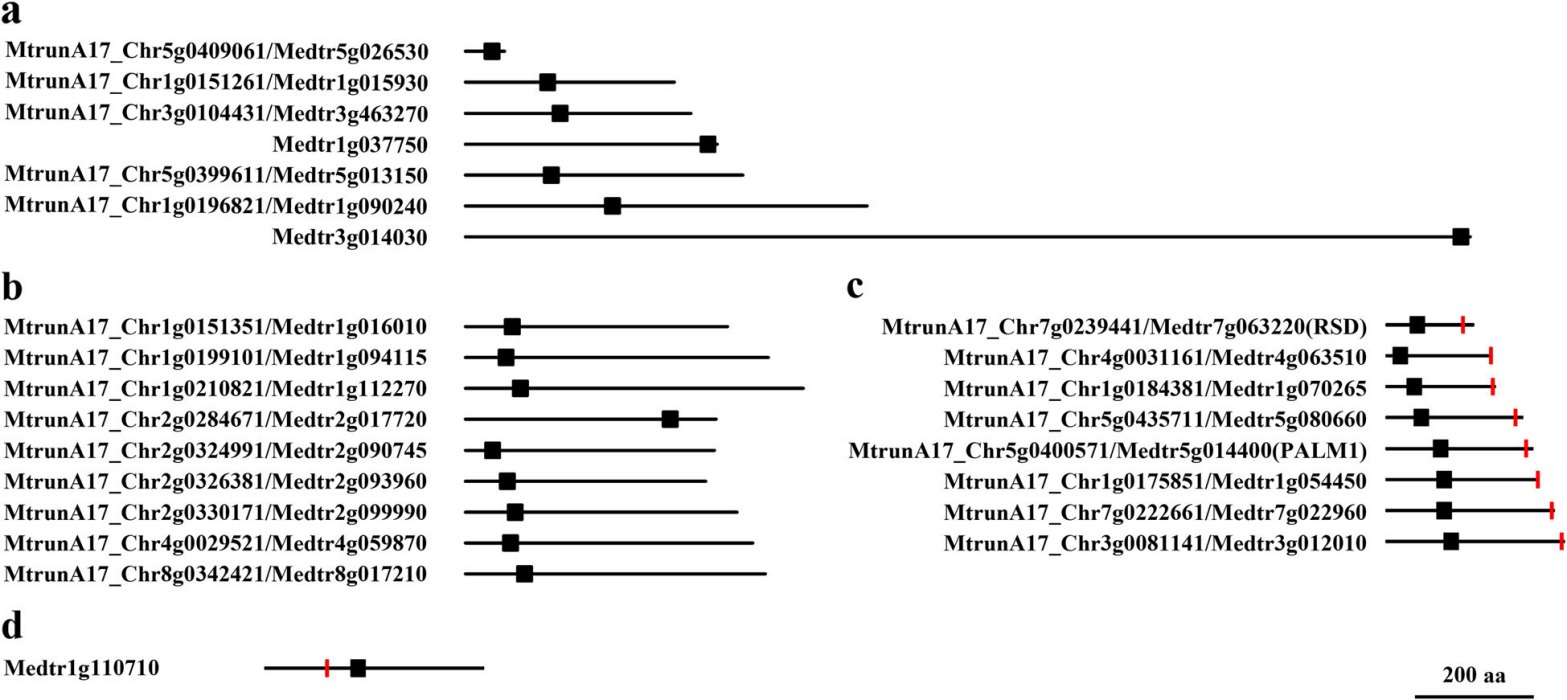
Representative single C2H2 ZFP in *M. truncatula*. **a** Single C2H2 ZFP without the EAR motif. **b** IDD homologs. **c** PALM1 and RSD type C2H2 ZFPs. The black line represents the protein sequence. The black and red rectangles indicate C2H2 and EAR motifs, respectively

The 74 single C2H2 ZFPs without an EAR motif varied significantly in length, ranging from 69 to 1890 amino acids (Fig. 4a). The distribution of the C2H2 motif was also variable, from the very N-terminus to the end of the C-terminus (Fig. 4a). These features highlight the sequence divergence of this subgroup, and imply the functional diversification among members. Unfortunately, none of the members have been characterized to date. To better understand the putative functions of the genes in this subgroup, we performed a sequence analysis and homolog search in other species. The resultant gene annotation showed that the function of this subgroup was significantly diversified. Some members were putative zinc finger transcription factors or C2H2-like zinc finger proteins, such as MtrunA17_Chr1g0151261/Medtr1g015930 and MtrunA17_Chr3g0104431/Medtr3g463270; some members were annotated as a helicase domain-containing protein such as Medtr3g014030 and Medtr1g037750; and some were predicted as the VEFS-Box of polycomb proteins, such as MtrunA17_Chr1g0196821/Medtr1g090240 and MtrunA17_Chr5g0399611/Medtr5g013150 (Fig. 4a). Fifteen IDD-type C2H2 motifs were found in this subgroup (Fig. 4b), which was highly similar to Arabidopsis IDD genes, suggesting that the members containing the IDD-type C2H2 motifs in *M. truncatula* might have similar functions to their Arabidopsis homologs.

MtrunA17_Chr8g0355141/Medtr8g043980, with a deduced length of 746 resides, is highly similar to Arabidopsis SERRATED LEAVES (SE, AT2G27100) [41], suggesting that it may be the *M. truncatula* homolog of SE. Arabidopsis PCF11P-SIMILAR PROTEIN 4 (PCFS4, AT4G04885) belongs to the C2H2 ZFPs gene family and plays a role in regulating flowering time [42]. The *M. truncatula* PCFS4 homolog has not yet been identified. We found that a member of this subgroup, MtrunA17_Chr6g0452881/Medtr6g011450, with a C2H2 motif located at the C-terminus, was highly similar to Arabidopsis PCFS4 and shared comparable C2H2 motif arrangement. This result suggests that MtrunA17_Chr6g0452881/Medtr6g011450 is probably the homolog of PCFS4.

As for the 60 single C2H2 ZFPs containing a single EAR motif, 59 of them had the C2H2 motif located upstream and EAR downstream. The C2H2 zinc finger was close to the N-terminus, whereas the EAR is placed at the far end of C-terminus, as represented by the reported PALM1 and RSD. This subgroup of C2H2 ZFPs was typically short, with 50 (83%) members less than 350 amino acids in length (Fig. 4c).

Except for PALM1 (MtrunA17_Chr5g0400571/Medtr5g014400) and RSD (MtrunA17_Chr7g0239441/Medtr7g063220), all the genes in this subgroup have not been characterized. By similarity analysis, we identified several genes that are very similar to *Arabidopsis* characterized C2H2 ZFPs. MtrunA17_Chr1g0175851/Medtr1g054450, which carried one C2H2 at N-terminus and EAR at C-terminus, appeared to be a close homolog of Arabidopsis ZFP8 (AT2G41940), soybean ZFP gene Glyma.09G107400 and rice C2H2 ZFP LOC_Os08g36110. MtrunA17_Chr5g0435711/Medtr5g080660 and MtrunA17_Chr1g0184381/Medtr1g070265 are very similar to Arabidopsis RABBIT EARS (REB, AT5G06070) [43] and SUPERMAN (SUP, AT3G23130) [19], respectively. Consistently, all these homologs had a similar motifs structure, i.e., one C2H2 and one EAR located at comparable positions (Fig. 4c, Additional file 10).

Medtr1g110710 is the only single C2H2 ZFPs with an EAR motif located upstream of the C2H2 zinc finger (Fig. 4d, Additional file 10). Medtr1g110710 is highly similar to and appears to be the homolog of Arabidopsis JAGGED (JAG, AT1G68480), which plays an important role in shaping lateral organs and promoting leaf tissue development [44, 45]. Sequence analysis also revealed that Medtr1g110710 was highly similar to soybean Glyma.10G273800, rice STAMENLESS 1 (SL1, LOC_Os01g03840) and a few other closely related genes in other plant species that share a similar gene structure to Medtr1g110710. SL1 has been reported to be the homolog of JAG [46]. Instead of regulating leaf development, SL1 controls floral development, demonstrating the functional divergence of the homologs [46]. Taken together, JAG, Medtr1g110710, and other homologs may represent a special cluster of C2H2 type ZFPs with an EAR motif upstream of the C2H2 zinc finger.

There were 17 single C2H2 ZFPs containing multiple EARs, including 16 genes containing two EARs and one gene containing three EARs (Additional file 10). The 16 ZFPs containing two EARs included nine ZFPs with the C2H2 motif placed between the two EARs, and another seven ZFPs with the C2H2 motif located downstream or upstream of the EARs (Additional file 11).

### ZFPs with multiple C2H2 motifs in *M. truncatula*

In the *M. truncatula* genome, 67 ZFPs harbored multiple C2H2 motifs, including 38 ZFPs containing two C2H2s, 16 ZFPs containing three C2H2s, 9 ZFPs containing four C2H2s, MtTRM1 (five C2H2s), 2 ZFPs containing six C2H2s and MtTFIIIA (nine C2H2s). ZFPs with multiple C2H2s have been widely found in other plant species [1, 15, 16, 27]. Based on the spacer length between motifs, the organization of C2H2s can be classified into dispersedly distributed motifs or tandem arrays [16, 27]. Using this method, we classified the 67 ZFPs with multiple C2H2s into two subgroups, i.e., 15 ZFPs contained tandem C2H2s and 52 ZFPs lacked tandem C2H2s.

**Fig. 5:**
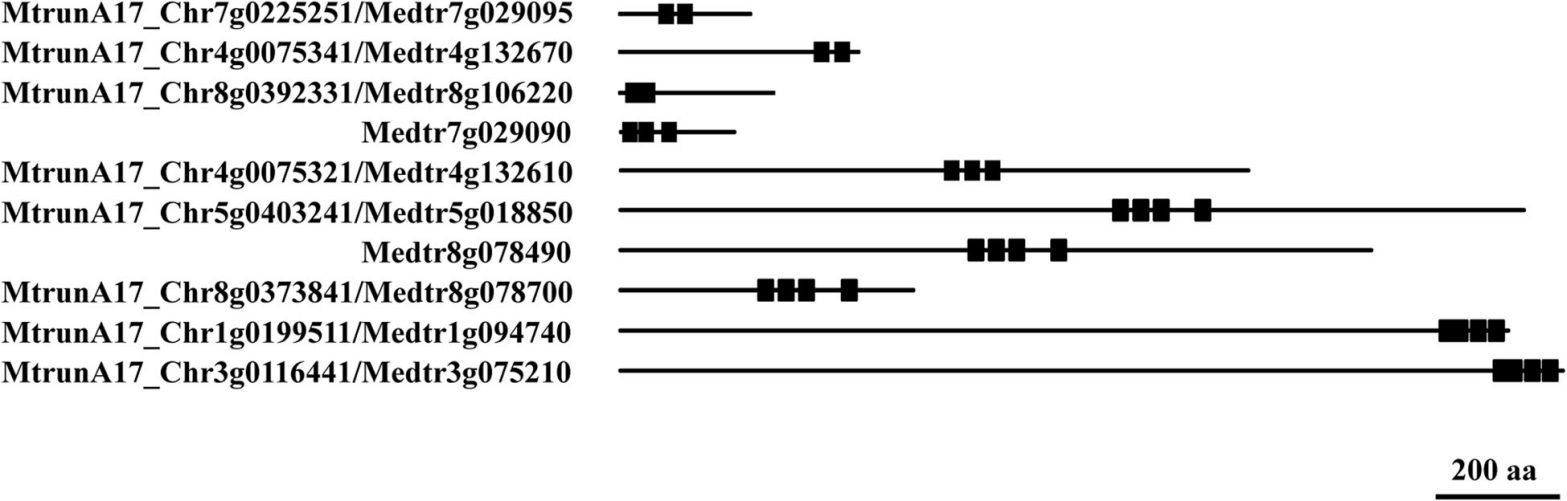
ZFPs with an array of C2H2 motifs in *M. truncatula*. The black line represents the protein sequence and the black rectangle indicates C2H2 motifs

The 15 ZFPs with tandem arrays of C2H2 motifs included three ZFPs with two C2H2s, two ZFPs with three C2H2s, six ZFPs with four C2H2s, and two ZFPs with six C2H2s, together with MtTRM1 and MtTFIIIA (Fig. 5). The spacer between C2H2 motifs in an array varied from zero to 11 residues. In MtrunA17_Chr8g0392331/Medtr8g106220, there were no link residues between two C2H2s, resulting in two continuous C2H2 motifs at the N-terminus (Fig. 5). The links between two C2H2s in MtrunA17_Chr7g0225251/Medtr7g029095 and MtrunA17_Chr4g0075341/Medtr4g132670 consisted of 5 and 10 residues, respectively (Fig. 5). Medtr7g029090 and MtrunA17_Chr4g0075321/Medtr4g132610, which had three C2H2s, showed a different array pattern. In MtrunA17_Chr4g0075321/Medtr4g132610, the links consisted of both 10 residues and three C2H2s forming a continuous tandem array. By contrast, the third C2H2 in Medtr7g029090 was isolated from the array formed by the first and second C2H2 (Fig. 5).

Among the nine ZFPs with four C2H2s, three of them (MtrunA17_Chr5g0403241/Medtr5g018850, Medtr8g078490, and MtrunA17_Chr8g0373841/Medtr8g078700) had the same array pattern. The first three C2H2s were tandemly linked by 10 or 9 residues, and the fourth C2H2 was isolated from the array. Notably, all three proteins exhibited similarities to Arabidopsis SU (VAR) 3–9-RELATED PROTEIN 5 (SUVR5, AT2G23740). Two of them, MtrunA17_Chr1g0199511/Medtr1g094740 and MtrunA17_Chr3g0116441/Medtr3g075210 are highly similar to Arabidopsis EARLY FLOWERING 6 (ELF6, AT5G04240) and RELATIVE OF EARLY FLOWERING 6 (REF6, AT3G48430), respectively. Despite the functional divergence, ELF6 and REF6 were highly similar to each other in sequence, and they both encoded Jumonji-class transcription factors containing four tandem C2H2 motifs at the C-terminus [47]. Similar to ELF6 and REF6, MtrunA17_Chr1g0199511/Medtr1g094740 and MtrunA17_Chr3g0116441/Medtr3g075210 also had an array of four C2H2 motifs located at the C-terminus, including a degraded C2H2, which has also been found in ELF6 and REF6 [16]. MtrunA17_Chr1g0199511/Medtr1g094740 and MtrunA17_Chr3g0116441/Medtr3g075210 are highly similar to soybean Glyma.20G181000 and Glyma.04G191900, the C2H2 arrays which have recently been reported [27].

For the 52 ZFPs without a tandem C2H2 array, the C2H2 zinc fingers were all separately located. To date, none of them has been characterized. A homolog search showed that three of them (MtrunA17_Chr1g0207451/Medtr1g106730, MtrunA17_Chr1g0152661/Medtr1g018420, and MtrunA17_Chr3g0134711/Medtr3g102980) had sequence similarity to Arabidopsis SALT TOLERANCE ZINC FINGER (STZ/AT10 AT1G27730) [48], suggesting that three ZFPs might mediate salt tolerance in *M. truncatula*. In addition, two members from a local tandem duplication, MtrunA17_Chr4g0049671/Medtr4g093260 and MtrunA17_Chr4g0049701/Medtr4g093270, exhibited similarity to members of the Arabidopsis HVA22 family, suggesting that they might also be involved in the stress response in *M. truncatula*.

### Expression analysis of *M. truncatula* C2H2 ZFPs

**Fig. 6:**
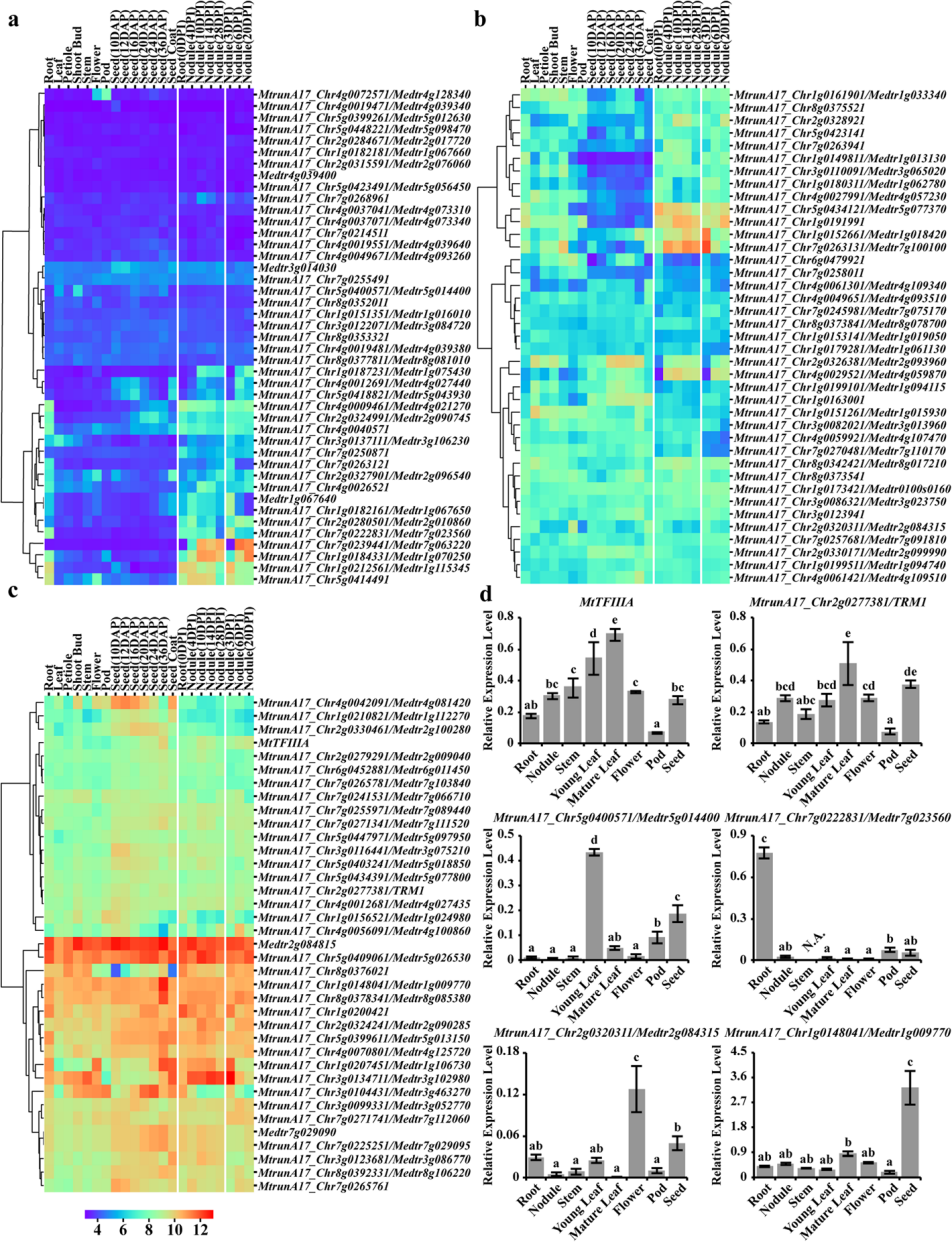
Tissue-specific expression analysis of C2H2 ZFPs in *M. truncatula*. **a** - **c** The transcript abundances of the C2H2 ZFPs showed by heatmaps generated from the *M. truncatula* Gene Expression Atlas data. **a** shows the genes with low transcript abundances, (**b**) shows the genes with medium transcript abundances, and (**c**) shows the genes with high transcript abundances. **d** The qRT-PCR result of six selected C2H2 genes. Shown are means ± standard deviations for three biological replicates and three technical replicates of each biological replicate. The relative expression levels of tested genes were normalized by the geometric mean of three endogenous control genes. ‘N.A.’ indicated undetectable expression. The lowercase letters above the bar indicate significant differences (*P* < 0.05) using ANOVA and Tukey’s test among samples

To understand the spatial expression patterns of *M. truncatula* C2H2 ZFPs, we analyzed the tissue-specific expression datasets for the C2H2 ZFPs in *M. truncatula*. By sequence comparison between the transcripts of C2H2 ZFPs and *M. truncatula* Affymetrix microarray probesets, we found that 119 out of a total of 218 ZFPs had the corresponding microarray probesets, enabling us to perform further expression analysis on *M. truncatula* Gene Expression Atlas (https://mtgea.noble.org/v3/) (Fig. 6a, b, c) [49, 50].

The C2H2 ZFPs exhibited a highly variable spatial expression pattern in general, suggesting that they might play different roles in regulating tissue development in *M. truncatula*. Eleven genes, including *MtrunA17_Chr4g0019471/Medtr4g039340*, *MtrunA17_Chr5g0399261/Medtr5g012630, MtrunA17_Chr5g0448221/Medtr5g098470, MtrunA17_Chr1g0182181/Medtr1g067660*, and *MtrunA17_Chr5g0423491/Medtr5g056450*, were not significantly detected in all tissues, implying they might play a minor role in regulating tissue development (Fig. 6a). By contrast, *Medtr2g084815*, *MtrunA17_Chr5g0409061/Medtr5g026530*, *MtrunA17_Chr1g0148041/Medtr1g009770* and *MtrunA17_Chr8g0378341/Medtr8g085380* showed high expression levels in all tissues examined (Fig. 6c). We also detected a few genes that were specifically expressed in certain tissues. For instance, *MtrunA17_Chr7g0222831/Medtr7g023560* showed extremely low expression in all tissues except for root and nodule, where it was significantly expressed (Fig. 6a). *MtrunA17_Chr5g0418821/Medtr5g043930* only showed expression in maturing seed, seed coat, and nodule, suggesting it might be involved in seed maturation and nodulation (Fig. 6a). Consistent with a previous report, *PALM1* (*MtrunA17_Chr5g0400571/Medtr5g014400*) only showed expression in leaf, shoot bud, and seed (Fig. 6a).

**Fig. 7:**
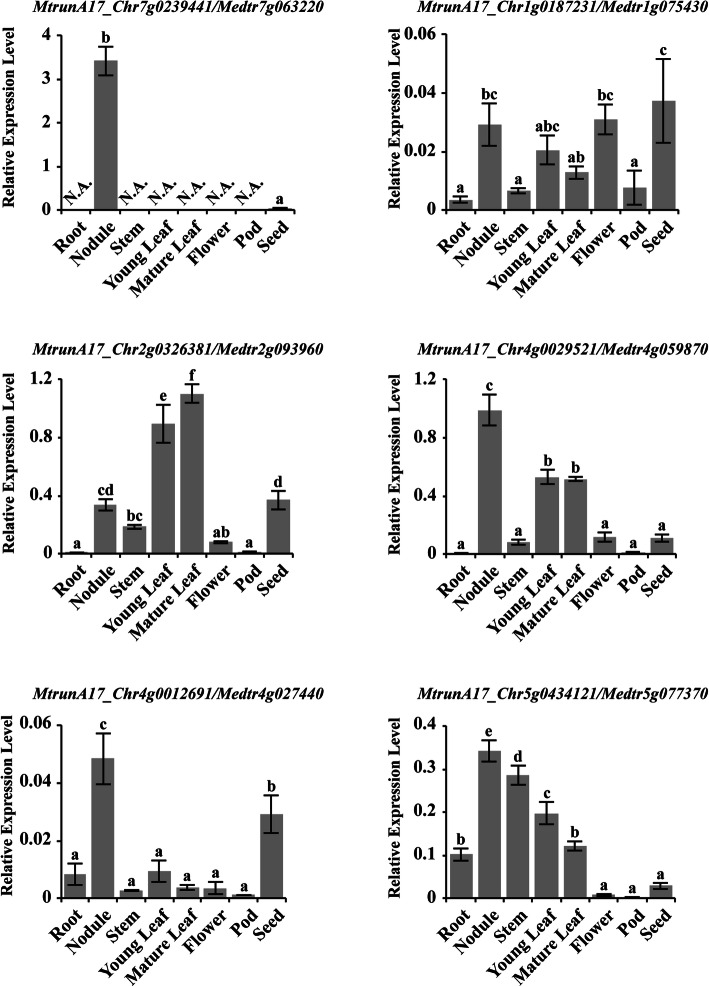
Confirmation of C2H2 ZFPs specifically expressed in *M. truncatula* symbiotic nodules. The qRT-PCR result of six selected C2H2 genes. Shown are means ± standard deviations for three biological replicates and three technical replicates of each biological replicate. The relative expression levels of tested genes were normalized by the geometric mean of three endogenous control genes. ‘N.A.’ indicated undetectable expression. The lowercase letters above the bar indicate significant differences (*P* < 0.05) using ANOVA and Tukey’s test among samples

To verify the expression pattern of these C2H2 ZFPs, we examined the expression of 25 genes using qRT-PCR (Fig. 6d, Fig. 7 and Additional file 12). The result indicated that *TFIIIA* and *TRM1* showed high-level expression in all tissues, whereas the other genes such as *MtrunA17_Chr5g0400571*/*Medtr5g014400, MtrunA17_Chr7g0222831/Medtr7g023560, MtrunA17_Chr2g0320311/Medtr2g084315* and, *MtrunA17_Chr1g0148041/Medtr1g009770* showed relatively high expression level in certain tissues (Fig. 6d, Fig. 7 and Additional file 12). Though the expression pattern varied for the select genes, they showed consistency with *M. truncatula* Gene Expression Atlas result, confirming the variable spatial expression pattern of the identified C2H2 ZFPs.

### Identification of C2H2 ZFPs specifically expressed in rhizobium symbiosomes

The legume symbiotic nodule fixed approximately 200 million tons of nitrogen and thus played a vital role in sustainable agriculture [51–53]. To identify ZFPs that were specifically expressed in *M. truncatula* symbiotic nodules, we analyzed the time course microarray datasets of *M. truncatula* root nodules for the C2H2 ZFPs. Among 119 ZFPs with corresponding probesets, fourteen showed obvious induction in nodules, including nine ZFPs with a single C2H2 motif, two with two C2H2 motifs, two with three C2H2 motifs and one with four C2H2 motifs. In strong support to a previous report [28], *RSD* (*MtrunA17_Chr7g0239441/Medtr7g063220*) was the ZFP that showed the strongest induction in nodules (Fig. 6a). Like *RSD*, *MtrunA17_Chr1g0187231/Medtr1g075430*, which contains a single C2H2 motif at the N-terminus and EAR motif at the C-terminus, also showed significant induction in nodules (Fig. 6a). MtrunA17_Chr2g0326381/Medtr2g093960 and MtrunA17_Chr4g0029521/Medtr4g059870, both containing a single C2H2 motif, are highly similar to the Arabidopsis BIRD family member BALDIBIS (BIB) and strongly expressed in nodules (Fig. 6b). BIB promotes tissue specification in Arabidopsis root by the transcriptional regulation of *SHR* [54]. As nodule development involves tissue specification, it is possible that MtrunA17_Chr2g0326381/Medtr2g093960 and MtrunA17_Chr4g0029521/Medtr4g059870 might play an important role in nodule development. *MtrunA17_Chr5g0434121/Medtr5g077370* was expressed in nodule, as well as vegetative tissues such as leaf, petiole, and shoot bud (Fig. 6b). Interestingly, four ZFPs that were induced in nodules, MtrunA17_Chr2g0326381/Medtr2g093960, MtrunA17_Chr4g0029521/Medtr4g059870, *MtrunA17_Chr4g0012691/Medtr4g027440*, and *MtrunA17_Chr5g0418821/Medtr5g043930*, also showed strong expression in seeds or seed coats, implying that they might regulate some common pathways in both nodule and seed (Fig. 6a, b).

To verify the nodule-specific expression pattern of C2H2 genes, we performed the qRT-PCR assay for six genes (Fig. 7). *RSD* (*MtrunA17_Chr7g0239441/Medtr7g063220*) showed extremely specific expression in nodule, while no signal was detected in other tissues. The rest five genes, *MtrunA17_Chr1g0187231/Medtr1g075430*, *MtrunA17_Chr2g0326381/Medtr2g093960*, *MtrunA17_Chr4g0029521/Medtr4g059870*, *MtrunA17_Chr4g0012691/Medtr4g027440* and *MtrunA17_Chr5g0434121/Medtr5g077370* showed high expression in nodule, though they also showed more or less expression in other tissues. Nevertheless, the expression pattern detected by qRT-PCR showed a good consistency with the Gene Atlas Expression result.

## Discussion

C2H2 zinc gene families have been summarized in several plant species, including Arabidopsis, rice, and poplar. Previous reports have shown that there are 176 C2H2 ZFPs in the Arabidopsis genome and 189 C2H2 ZFPs in both rice and poplar [1, 15, 16, 27]. In the important legume species soybean, 321 C2H2 ZFPs have been identified [27]. In this work, we identified 218 C2H2 type zinc finger proteins bearing 337 individual C2H2 motifs in the model leguminous species *M. truncatula*. Both belonging to papilionoids, *M. truncatula* and soybean are closely related and shared a WGD dated approximately 58 million years (Myr) ago [35, 37, 55]. Soybean underwent another WGD 13 Myr ago that resulted in a paleopolyploid genome, in which nearly 75% of genes have duplicated copies, explaining why more C2H2 ZFPs were identified in the soybean genome. In *M. truncatula*, local duplication of genes has been reported in many gene families, such as the heat shock transcription factor family and the F-box gene family [56–58]. Genome sequencing data have shown that *M. truncatula* has very high rates of local gene duplications that have significantly contributed to the expansion of gene families [35]. This is probably the reason why we detected a large number of C2H2 ZFPs. As the evidence, we have found local gene duplication events that resulted in locally duplicated C2H2 ZFPs.

We classified 337 individual C2H2 motifs into five major groups, Q-type, IDD type, QM-type, Z-type, and C-type. Though Q-type C2H2s have been widely found in plants, the conserved residues between the two histidines have not been described yet. In this work, we identified certain conserved patterns of the residues, such as the consensus ‘QNA’, between the two histidines of the Q-type C2H2 zinc fingers. Although the function of the residues between the histidines is yet to be understood, it is highly likely that they may contribute to the functional diversification of the Q-type C2H2 motifs. Thus, it was reasonable to further group Q-type C2H2 motifs into subgroups based on the conservation of residues between two histidines. The core sequence of Q-type can be altered, resulting in a modified Q-type (QM-type), which contained 57 members in *M. truncatula*. We have revealed that the C2H2 motifs from IDD genes displayed a unique conservation pattern that distinguished them from the family, and thus we named them IDD-type C2H2 fingers. Z-type C2H2 fingers refer to the motifs that show a certain pattern of conservation for residues other than cytosine and histidine. Due to insufficient studies regarding the function of Z-type C2H2s, we currently are unable to provide a functional explanation for the subgroups within Z-type C2H2s.

EAR is a transcriptional repression motif and enables the repression role to the transcription factor. We also found a wide presence of the EAR motif in single C2H2 motif ZFPs, typically at the C-terminus of the ZFPs, as represented by PALM1/IRG1 and RSD. We also found a putative EAR motif located in the fifth C2H2 motif of TRM1 from *M. truncatula* and other plant species. TRM1 is known as a transcription repressor. The identification of a putative EAR motif in TRM1 homologs is very interesting, although further experimental verification is needed to address the role of this putative motif.

First identified in *Xenopus laevis*, TFIIIA has been identified in plant species as the ZFP containing the most C2H2 zinc fingers in plants [16, 34, 59, 60]. Given the essential role in regulating 5S rRNA transcription, it is highly likely that *M. truncatula* harbors the homolog of TFIIIA. Initially, we did not find TFIIIA homolog in *M. truncatula* genome database. By a comprehensive approach including analyzing the Affymetrix probesets, we successfully identified TFIIIA homolog in *M. truncatula*. In addition, we cloned and sequenced the coding sequence of *M. truncatula* TFIIIA homolog. The gene structure and arrangement of C2H2 motifs display high conservation among the TFIIIA homologs from higher plant species, suggesting their conserved function and evolutionary history in plants. We cloned and analyzed TRM1 in *M. truncatula*, and identified TRM1 from other higher plant species. TRM1 was first cloned in maize as a YY1-like transcription repressor with a specific role in suppressing *rbcS-m3* in mesophyll cells. The conserved sequences and motif arrangement of the TRM1 homolog in *M. truncatula* and other plants indicated that TRM1-mediated suppression of *rbcS-m3* might be highly conserved in higher plants.

Although the C2H2 motifs demonstrate high sequence similarity, the entire protein sequences of C2H2 ZFPs display very low similarity across the family. Due to its capacity to bind DNA, the C2H2 motif has been adopted by many transcription factors with very distinct functions to recognize the cis-elements. The varied sequence outside the C2H2 motif may reflect the functional diversification of ZFPs in the family. In support of this notion, the expression pattern revealed by gene atlas analysis and qRT-PCR showed that ZFPs exhibit significant variations in a tissue-specific expression pattern.

## Conclusions

In summary, we identified 218 C2H2 ZFPs with 337 C2H2 motifs in the model legume *M. truncatula*. Based on the quantity and arrangement of C2H2 motifs and EAR motifs, we classified these ZFPs into different groups. Using similarity-based analysis, we found many important genes in the identified C2H2 ZFP gene family in *M. truncatula*. We also analyzed the sequence of C2H2 motifs and identified a new type of C2H2 motif, IDD type of C2H2. Additionally, we identified and cloned the TFIIIA and TRM1 homolog in the *M. truncatula* genome. Expression analysis showed that the identified C2H2 ZFPs can be clustered into three groups according to their transcript abundance in different tissues. We also found and confirmed that several C2H2 ZFP genes are specifically expressed in certain tissues, such as flower, seed, and nodule. Our genome-wide work provided new insights into the diversification and expansion of C2H2 ZFPs in higher plants.

## Methods

### Plant materials

The seeds used in this study were obtained from the stock center of the Department of Grassland Science of South China Agricultural University. The seeds of *M. truncatula* (Jemalong A17 and R108) were rubbed on sandpapers and germinated on moist filter papers. After germination, the plants were grown in a greenhouse for further experiments.

### RNA extraction and qRT-PCR

Total RNA was extracted from roots, stems, unopened young leaves, mature leaves, flowers, immature pods, immature seeds and 16 days post-inoculation (dpi) nodules of Jemalong A17 using TRIzol Reagent (Invitrogen, 15,596,026) and Quick RNA isolation Kit (Huayue yang Biotech, 0416–50) according to the manufacturers’ instructions. The reverse transcription was performed with TransScript One-Step gDNA Removal and cDNA Synthesis SuperMix (Transgen, AT311–03) using 1.6 μg total RNA. The qRT-PCR was performed with ChamQ™ Universal SYBR qPCR Master Mix (Vazyme, Q711–02) on Bio-rad CFX Connect™ Real-Time System. Three biological replicates and three technical replicates of each biological replicate were used for qRT-PCR. Relative expression level of tested genes was normalized using the geometric mean of *MtActin* (*MtrunA17_Chr3g0129641/ Medtr3g095530*) *MtGAPDH* (*MtrunA17Chr3g0122971*/*Medtr3g085850*) and *MtHELICASE* (*MtrunA17Chr4g0077061* /*Medtr4g134790*) three genes [61–63]. The statistical significance between samples was examined by One-way ANOVA and Tukey’s test at the *P* = 0.05 level in SPSS 13.0 software (https://www.ibm.com/analytics/spss-statistics-software). The qRT-PCR primers are listed in the supplementary material (Additional file 13).

### Identification of C2H2 ZFPs

There are only 112 *M. truncatula* C2H2 ZFPs in the Plant Transcription Factor Database (http://planttfdb.cbi.pku.edu.cn/index.php?sp=Mtr) and 77 C2H2 type ZFPs in the *M. truncatula* Gene Expression Atlas (https://mtgea.noble.org). Given the genome size and C2H2 ZFP gene number in other plant species, we thought there should be more C2H2 ZFPs in the *M. truncatula* genome. To extensively identify C2H2 ZFPs in the *M. truncatula* genome, we downloaded and analyzed the two versions of *M. truncatula* genome sequences, MtrunA17r5.0 (https://medicago.toulouse.inra.fr/MtrunA17r5.0-ANR) and Mt4.0v2 (http://www.medicagogenome.org) [31–33]. A python script (Python 3.6) was developed to search the annotated proteins. In the script, the regular expression (https://docs.python.org/3/library/re.html) [64] “.{2} C.{2} C.{12} H.{3,5} H” or “.{2} C.{4} C.{12} H.{3,5} H” was used to match the C2H2 motifs. The candidate C2H2 ZFPs from this step were further scanned at PROSITE (https://prosite.expasy.org) and PFAM (https://pfam.xfam.org) platforms for confirmation. The candidates that passed the confirmation were considered as the C2H2 type ZFPs. A custom Python script was developed to draw the gene structure and arrangement of C2H2 motifs in the genes.

### Splice variant and physicochemical property analysis of C2H2 ZFPs

The splice variants of C2H2 ZFPs were marked according to the annotation of *M. truncatula* Mt4.0v2 genome database [32, 33]. The physicochemical property of the proteins was calculated with the ExPASy Compute pI/Mw tool (http://web.expasy.org/compute_pi).

### Identification of TFIIIA and TRM1 homolog in *M. truncatula*

To identify *M. truncatula* TFIIIA and TRM1 homologs, Arabidopsis TFIIIA and maize TRM1 protein sequences were used as queries to search against the *M. truncatula* protein databases (Mt4.0v2 and MtrunA17r5.0) by BLAST at the cutoff e-value of 1e-15 [65]. Unfortunately, the BLAST program did not return positive results for TFIIIA, but the *MtrunA17_Chr2g0277381* was found as the homolog of AtTRM1 in MtrunA17r5.0 protein database instead of Mt4.0v2. Next, we used the same query sequences of TFIIIA to search the *M. truncatula* R108 genome sequences database and *M. truncatula* Affymetrix probesets. The TBLASTN results indicated that a region located on scaffold MWMB01000024 showed very high similarity to the Arabidopsis TFIIIA. Two *M. truncatula* probesets, *Mtr.14440.1.S1_at* and *Mtr.25069.1.S1_s_at*, showed very high similarity to the Arabidopsis TFIIIA and maize TRM1, respectively. Next, we further cross-searched the *M. truncatula* R108 genome sequences and probesets. The results indicated that the probesets *Mtr.14440.1.S1_at* corresponded to the fragment on scaffold MWMB01000024 that showed sequence similarity to TFIIIA, and the probesets *Mtr.25069.1.S1_s_at* corresponded to the locus on chromosome 2 (*MtrunA17_Chr2g0277381*) that showed sequence similarity to maize TRM1. Based on the results above, we annotated the *M. truncatula TFIIIA* orthologues. To identify the TFIIIA and TRM1 homologs from other species, we used MtTFIIIA and MtTRM1 protein sequences as queries and searched Phytozome database (https://phytozome.jgi.doe.gov/pz/portal.html#). The genes with high similarity to MtTFIIIA and MtTRM1 were downloaded for the sequence and motif analysis. We also used the TFIIIA and TRM1 homologs from other species to build an HMM profile and searched the protein database of *M. truncatula* by HMMER (http://hmmer.org/). The result confirmed the identified MtTFIIIA and MtTRM1were homologs of TFIIIA and TRM1.

### cDNA cloning and sequencing

The cDNA of *M. truncatula* R108 or A17 leaves was used for the cloning of *MtTFIIIA* and *MtTRM1*, respectively. The coding sequences of *MtTFIIIA* and *MtTRM1* were amplified using Q5® High-Fidelity DNA Polymerase (NEB #M0491) by a standard PCR protocol as follows: 98 °C for 10 s, 60 °C for 30 s and 72 °C for 45 s for 35 cycles. The PCR products of coding sequences were Sanger sequenced. The PCR primers are listed in the supplementary material (Additional file 13).

### Phylogenetic analysis

The protein sequences of TFIIIA, TRM1 homologs, or other C2H2 ZFPs were aligned by ClustalX program (www.clustal.org) [66]. For the multiple protein sequences alignment, Gonnet series was used as the Protein Weight Matirx, the Gap Opening was set to 10.00, the Gap Extention was set to 0.20, and the Delay Divergent Sequences was set to 30%. The Maximum Likelihood method based on the Dayhoff matrix was used to reconstruct the phylogenetic tree in MEGA 7.0 software [67]. The bootstrap replicates were set to 1000.

### Motif classification and analysis

To classify the C2H2 motifs, the motif sequences were aligned using ClustalX program (www.clustal.org) [66]. By manual observation and comparison with a previous method [12, 14], C2H2 motifs were classified based on the core sequence (Additional file 8). The C2H2 arrays are defined as C2H2s with a link of sequence fewer than 11 residues [15].

### Classification and chromosomal localization of C2H2 ZFPs in *M. truncatula*

The C2H2 ZFPs were naturally classified into groups based on the number of C2H2 motifs in the proteins. The chromosomal coordination of the identified C2H2 ZFPs was pulled out from the *M. truncatula* genome annotation file. The MapChart 2.32 program was used to generate the diagram illustrating the chromosomal localization of C2H2 ZFPs in *M. truncatula* [68].

### Identification of local gene duplication and gene pairs result from WGD

We used a previously reported method to identify the local gene duplication in *M. truncatula* [35]. Briefly, the C2H2 genes located within 100 adjacent gene models were treated as local gene duplication. MCScanX program was used to identify the C2H2 gene pairs resulted from WGD [38]. Briefly, all proteins of *M. truncatula* were searched against the *M. truncatula* protein database. The result was further analyzed by MCScanX ‘duplicate_gene_classifier’ code to identify the gene pairs from WGD. The identified gene pairs were also further confirmed by searching the previously published gene pairs datasets [35].

### Expression analysis

All C2H2 ZFPs were first searched against the *M. truncatula* Affymetrix microarray probesets. For the ZFPs with corresponding probesets, the tissue level expression data were downloaded from the *M. truncatula* Gene Expression Atlas (MtGEA, https://mtgea.noble.org/v3) [48, 49]. A custom Python script was developed to construct the heat map illustrating the log2 transformed expression level of ZFPs.

## Supplementary information

**Additional file 1. **C2H2 ZFPs in *Medicago truncatula*. The list of gene ID, location, probeset, physicochemical property, splice variant, ortholog locus, motif, and protein sequence for each C2H2 ZFPs.

**Additional file 2.** The protein sequences of C2H2 ZFPs.

**Additional file 3.** The C2H2 motif sequences of C2H2 ZFPs.

**Additional file 4. **Distribution of C2H2 ZFPs on chromosomes in *M.truncatula*.

**Additional file 5. **Local gene duplications of C2H2 ZFPs in *M.truncatula*.

**Additional file 6. **C2H2 ZFP gene pairs originated from whole-genome duplication in *M.truncatula*.

**Additional file 7.** C2H2 ZFPs paired sequence similarity analysis.

**Additional file 8. **Classification of C2H2 motif in *M.truncatula*.

**Additional file 9. **Classification of C2H2 ZFPs in *M.truncatula*.

**Additional file 10.** EAR motif in C2H2 ZFPs with one zinc finger. ‘N.A.’ indicated that this C2H2 ZFP did not contain the EAR motif.

**Additional file 11. **Representative C2H2 ZFPs with a single C2H2 motif and two EAR motifs in *M. truncatula*. The black line represents the protein sequence. The black and red rectangles indicate C2H2 and EAR motifs, respectively.

**Additional file 12. **Tissue-specific expression analysis of fifteen C2H2 ZFPs by qRT-PCR in *M. truncatula*. The qRT-PCR result of thirteen selected C2H2 genes. Shown are means ± standard deviations for three biological replicates and three technical replicates of each biological replicate. The relative expression levels of tested genes were normalized by the geometric mean of three endogenous control genes. ‘N.A.’ indicated undetectable expression. The lowercase letters above the bar indicate significant differences (*P* < 0.05) using ANOVA and Tukey’s test among samples.

**Additional file 13.** The list of primers.

## Data Availability

The datasets used in this study are included in the Additional Tables. Genomic DNA, cDNA and protein sequences of *MtTFIIIA* and *MtTRM1* can be found in the NCBI GenBank under the accession numbers MT799184, MK830055, QJS95126, MT799185, MK830056, and QJS95127, respectively.

## Abbreviations

C2H2 ZFPs: Cys (2) His (2) zinc finger proteins
ZFPs: Zinc finger proteins
EAR: Ethylene-responsive element binding factor-associated amphiphilic repression motif
QM-type: Modified Q-type
qRT-PCR: Quantitative RT-PCR
WGD: Whole-genome duplication
Myr: Million years ago
IDD: Indeterminate-domain
