# Supplementary figures and images for: Genome-wide study of C2H2 zinc finger gene family in Medicago truncatula
Source: BMC Plant Biol. 2020 Aug 31;20:401. doi: 10.1186/s12870-020-02619-6 (PMC7460785; doi:10.1186/s12870-020-02619-6)

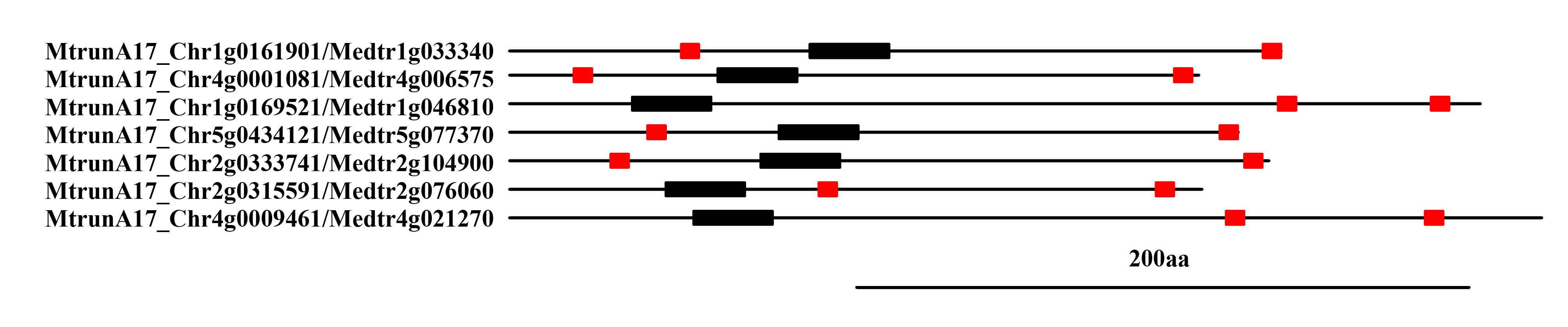

Supplement: Supplementary file 11 — Additional file 11. Representative C2H2 ZFPs with a single C2H2 motif and two EAR motifs in M. truncatula. The black line represents the protein sequence. The black and red rectangles indicate C2H2 and EAR motifs, respectively. [file 12870_2020_2619_MOESM11_ESM.tif]

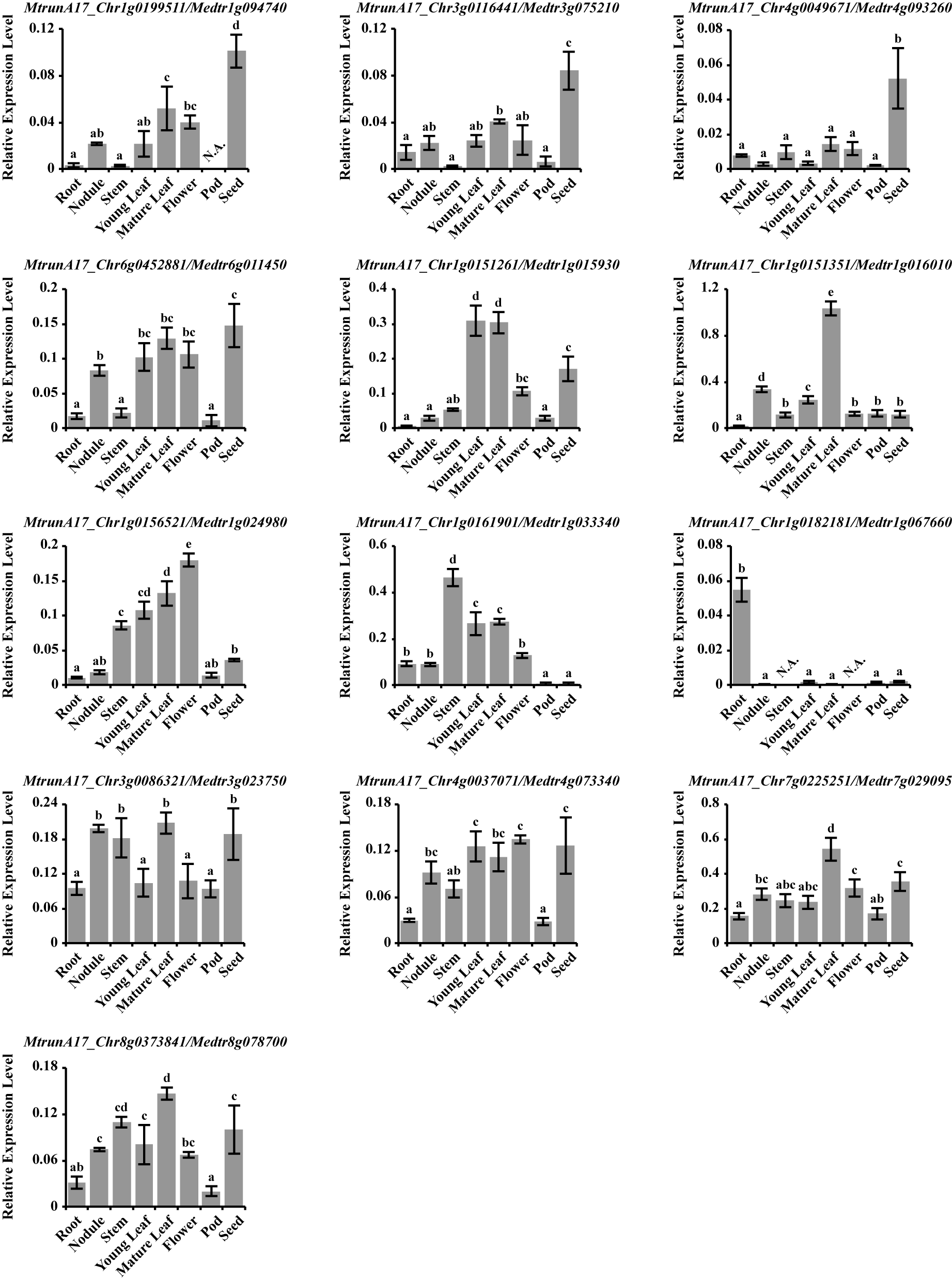

Supplement: Supplementary file 12 — Additional file 12. Tissue-specific expression analysis of fifteen C2H2 ZFPs by qRT-PCR in M. truncatula. The qRT-PCR result of thirteen selected C2H2 genes. Shown are means ± standard deviations for three biological replicates and three technical replicates of each biological replicate. The relative expression levels of tested genes were normalized by the geometric mean of three endogenous control genes. ‘N.A.’ indicated undetectable expression. The lowercase letters above the bar indicate significant differences (P < 0.05) using ANOVA and Tukey’s test among samples. [file 12870_2020_2619_MOESM12_ESM.tif]
